# Favorable effect of the combination of vinorelbine and dihydropyrimidine dehydrogenase-inhibitory fluoropyrimidine in *EGFR*-mutated lung adenocarcinoma: Retrospective and *in vitro* studies

**DOI:** 10.3892/ijo.2015.2815

**Published:** 2015-01-07

**Authors:** HIROKI IZUMI, HIROKAZU TOUGE, TADASHI IGISHI, HARUHIKO MAKINO, SHIZUKA NISHII-ITO, MIYAKO TAKATA, HIROFUMI NAKAZAKI, YASUTO UEDA, SHINGO MATSUMOTO, MASAHIRO KODANI, JUN KURAI, KENICHI TAKEDA, TOMOHIRO SAKAMOTO, MASAAKI YANAI, NATSUMI TANAKA, CHAITANYA S. NIRODI, EIJI SHIMIZU

**Affiliations:** 1 Division of Medical Oncology and Molecular Respirology, Faculty of Medicine, Tottori University, Yonago, Tottori 683-8504, Japan; 2 Department of Oncologic Sciences, Mitchell Cancer Institute, University of South Alabama, Mobile, AL 36604, USA

**Keywords:** vinorelbine, dihydropyrimidine dehydrogenase-inhibitory fluoropyrimidine, 5-fluorouracil, epidermal growth factor receptor, lung adenocarcinoma

## Abstract

Although cytotoxic chemotherapy is essential in epidermal growth factor receptor (*EGFR*)-mutated non-small cell lung cancer (NSCLC), it is unclear which regimen is most effective. We retrospectively compared the efficacy of standard platinum-based chemotherapy with that of combination chemotherapy using vinorelbine (VNR) plus dihydropyrimidine dehydrogenase-inhibitory fluoropyrimidine (DIF) in *EGFR*-mutated lung adenocarcinomas, and we investigated a potential mechanism by which the combination chemotherapy of VNR + DIF was favorable in the treatment of *EGFR*-mutated lung adenocarcinoma *in vitro*. In our retrospective analysis, the response rate and disease control rate afforded by the VNR + DIF treatment tended to be better than those by platinum-based chemotherapy, and the progression-free survival of the 24 VNR + DIF-treated patients was significantly longer than that of the 15 platinum-based chemotherapy patients. In *EGFR*-mutated PC9 cells, VNR induced EGFR dephosphorylation at a clinically achievable concentration. 1BR3-LR cells, a line of fibroblast cells transfected with a mutant *EGFR* construct, were completely resistant to gefitinib in the medium containing 10% fetal bovine serum (FBS), whereas the sensitivity of these cells to gefitinib was increased in 0.5% FBS-containing medium. Similarly, the sensitivity of 1BR3-LR cells to VNR was increased when they were cultured in low-serum condition. In addition, sodium orthovanadate (Na_3_VO_4_) inhibited the EGFR dephosphorylation induced by VNR or gefitinib and suppressed the cell growth inhibition by these agents in PC9 cells. VNR and gefitinib showed synergistic cell growth inhibition in combination with 5-fluorouracil (5-FU) in PC9 cells. We propose that the EGFR dephosphorylation induced by VNR is related to cell growth inhibitory activity of VNR, and that this is one of the mechanisms of the synergistic effect of VNR + 5-FU in *EGFR*-mutated lung cancer cells. In conclusion, the combination chemotherapy of VNR + DIF may be a promising treatment for NSCLC patients with *EGFR* mutations.

## Introduction

Lung cancer is the leading cause of cancer-related death worldwide. More than 80% of lung cancers are non-small cell lung cancers (NSCLCs), and lung adenocarcinoma is the most common type of NSCLC. The median survival of patients with metastatic NSCLC treated with cytotoxic chemotherapy agents is 10–12 months (1,2).

Epidermal growth factor receptor (EGFR), a member of the family of growth factor receptor tyrosine kinases, is expressed in a variety of solid cancers. *EGFR* somatic mutations were identified in 5–40% of NSCLCs, and is especially common in never-smokers, women, Asians, and patients with adenocarcinoma (3–6). NSCLCs harboring-activated *EGFR* mutations are addicted to EGFR signaling, and treatment with small-molecule EGFR-tyrosine kinase inhibitors (TKIs) such as gefitinib and erlotinib demonstrated dramatic responses to lung adenocarcinomas with *EGFR* mutations (7,8). However, almost all lung adenocarcinoma patients with *EGFR* mutations who respond to EGFR-TKIs ultimately develop resistance to these agents. Therefore, to prolong the survival time of advanced NSCLC patients with *EGFR* mutations, conventional cytotoxic chemotherapy is necessary regardless of whether it is administered before or after treatment with EGFR-TKIs.

At present, the combination of platinum with one of several chemotherapeutic agents [docetaxel, paclitaxel, gemcitabine, vinorelbine (VNR), irinotecan, pemetrexed or FT-5-chloro-2,4-dihydroxypyridine-potassium oxonate (S-1)] is considered a standard chemotherapy for advanced NSCLC (1,2,9,10). However, non-platinum combinations of third-generation drugs such as gemcitabine + VNR have less toxicity and almost equivalent efficacy compared to platinum-based chemotherapy (11). Therefore, non-platinum combination chemotherapy can be an option as a first-line treatment, even in patients with advanced NSCLC harboring *EGFR* mutations.

VNR, which is widely used to treat solid tumors such as NSCLC and breast cancer, is a semisynthetic vinca-alkaloid derived from vinblastine. This chemotherapeutic agent is one of the standard treatment agents for elderly patients with NSCLC (12), and, in combination with cisplatin, VNR is the only third-generation drug that demonstrated a consistent improvement of survival in the adjuvant setting of resected NSCLC (13–15).

UFT is an oral anticancer agent combining tegafur (FT) and uracil at a molar ratio of 1:4. FT is a prodrug of 5-fluorouracil (5-FU), and uracil is a competitive and reversible inhibitor of dihydropyrimidine dehydrogenase (DPD), the rate-limiting enzyme responsible for the catabolism of 5-FU. S-1 is a novel oral fluorouracil antitumor drug that combines FT, 5-chloro-2,4-dihydroxypyridine (which inhibits DPD activity), and potassium oxonate (which reduces gastrointestinal toxicity). UFT and S-1 are referred to as dehydrogenase-inhibitory fluoropyrimidine (DIF).

UFT is effective in prolonging the survival of patients with NSCLC after surgical resection (16,17). In a recent phase III trial, the combination chemotherapy of S-1 with carboplatin was not inferior in terms of overall survival (OS) compared with a standard chemotherapy, carboplatin and paclitaxel, for patients with advanced NSCLC (9). These results suggest the potential of DIF as a chemotherapeutic agent for advanced NSCLC.

We reported the schedule-dependent synergistic effect of VNR and subsequent 5-FU or UFT on NSCLC *in vitro* and in an animal model (18). Based on these preclinical data, we conducted two phase II studies of VNR + DIF, under which VNR was infused on days 1 and 8, and 600 mg/day UFT or 80 mg/m^2^/day S-1 was administered daily from day 2 to 6 and from day 9 to 13 in a 3-week cycle. The combination therapy of VNR + UFT was shown to be feasible and active in the treatment of elderly patients with advanced NSCLC (19). Promising results were also observed in unselected advanced NSCLC patients treated with the combination of VNR + S-1 (20).

In the process of clinical trials and clinical practice applying the combination treatment of VNR + DIF for advanced NSCLC, we noticed that patients exhibiting long-term stable disease tended to harbor *EGFR* mutations. This finding raised a hypothesis that the combination treatment of VNR + DIF may be specifically effective in NSCLC patients with *EGFR* mutations.

In the present study, we retrospectively compared the efficacy of the combination treatment of VNR + DIF with that of the standard platinum-based chemotherapy in patients with lung adenocarcinoma harboring *EGFR* mutations. We also sought to identify the mechanism by which the combination chemotherapy of VNR + DIF was more favorable than platinum-based chemotherapy in NSCLC harboring *EGFR* mutations *in vitro*.

## Materials and methods

### Comparison of the effects of chemotherapies

We retrospectively reviewed 39 lung adenocarcinoma patients harboring *EGFR* mutations who were diagnosed from November, 2004 to March, 2013 at Tottori University Hospital in Yonago, Japan and who received the combination therapy of VNR + DIF or platinum-based chemotherapy for the first cytotoxic chemotherapy. The presence of *EGFR* mutation was evaluated by the polymerase chain reaction (PCR)-invader method (BML, Inc., Tokyo, Japan). *EGFR* mutation analyses were not performed in four cases. These patients achieved long-term progression-free survival (PFS) times of >6 months with gefitinib treatment. The PFS was <6 months in >95% of the *EGFR* mutation-negative patients (21). Thus, we considered these four patients as *EGFR* mutation-positive cases.

The differences between the two groups were compared by the Mann-Whitney test and χ^2^ or Fisher’s exact test for numerical and categorized data, respectively. Tumor response was evaluated according to the Response Evaluation Criteria in Solid Tumors (RECIST) (22). The OS and PFS times following the first-line cytotoxic chemotherapy was assessed using the Kaplan-Meier method and compared by the log-rank test. P<0.05 was considered significant.

### Chemicals and reagents

VNR (Kyowa Hakko Kirin Co., Ltd., Tokyo, Japan) was dissolved in distilled water and stored at −20°C. A stock solution of cisplatin (CDDP) (Nippon Kayaku Co., Ltd., Tokyo, Japan) was reconstituted with water, diluted in 0.9% sodium chloride solution, and stored at −20°C. Gefitinib (AstraZeneca, Cheshire, UK) and 5-FU (KyowaHakko Kirin Co., Ltd.) were dissolved in dimethyl sulfoxide and stored at −20°C. 3-(4,5-Dimethylhiazol-2-yl)-2,5-diphenyltetrazolium bromide (MTT) (Wako Pure Chemical Industries, Ltd., Osaka, Japan) was dissolved in phosphate-buffered saline (PBS) and stored at −20°C.

### Cell lines and cultures

The human NSCLC cell line PC9, which harbors an *EGFR* exon 19 deletion mutation (ΔE746-A750) (23) was obtained from the RIKEN BioResource Center (Ibaraki, Japan). The fibroblast cell line 1BR3, stably transfected with a mutant *EGFR* construct with an L858R replacement in exon 21 (1BR3-LR), was a generous gift from Dr David J. Chen (24). The PC9 cells were maintained in RPMI-1640 medium supplemented with 10% fetal bovine serum (FBS) and antibiotics (100 U/ml penicillin and 100 μg/ml streptomycin). 1BR3-LR cells were maintained in MEM-α medium supplemented with 10% FBS and antibiotics (100 U/ml penicillin, 100 μg/ml streptomycin, and 2 μg/ml blasticidin). These cells were grown in a humidified atmosphere of 5% CO_2_/95% air at 37°C.

### MTT assay

The cell growth inhibition by chemotherapeutic agents was determined by an MTT assay. Cells counted with a hematocytometer were plated in 96-well flat-bottom multiplates (Nalge Nunc International Corp., Rochester, NY, USA) in 100 μl of medium and incubated overnight to permit cell attachment. The medium was then removed from each well and replaced with 100 μl medium containing the drugs for the indicated time. After 72 h, 10 μg of MTT in 10 μl PBS was added to each well, and incubation was continued for an additional 4 h. Thereafter, 100 μl of 0.04 N HCl in 2-propanol was added, and the multiplates were incubated overnight to solubilize the MTT formazan crystal. The absorbance of each well was measured at 570 nm wavelength (reference 650 nm) using a Tecan Sunrise scanning multiwell spectrometer (Tecan Japan Co., Ltd., Kanagawa, Japan). Each experiment was performed in triplicate for each drug concentration and was independently performed three times.

### Immunoprecipitation and western blot analysis

Cells were incubated in 6-well tissue culture plates overnight and washed with ice-cold PBS and lysed in lysis buffer [1% NP-40, 0.25% sodium deoxycholate, 150 mM NaCl, pH 7.4, 50 mM Tris-HCl, 1 mM EDTA, 1 mM NaF, 1 mM sodium orthovanadate (Na_3_VO_4_)] including 1 mM phenylmethylsulfonyl fluoride, 1 μg/ml leupeptin, 1 μg/ml aprotinin, and 1 μg/ml pepstatin. After 5 min on ice, lysates were centrifuged at 13,000 × g for 10 min at 4°C, and the supernatant was then collected. Protein was measured using the Bio-Rad Protein Assay reagent (Bio-Rad Laboratories, Hercules, CA, USA), and protein lysates containing 20 μg of total cellular protein or immunoprecipitates with the indicated antibodies were subjected to discontinuous sodium dodecyl sulfate-polyacrylamide gel electrophoresis (SDS-PAGE).

Proteins were electrotransferred to a polyvinylidene fluoride (PVDF) membrane (GE Healthcare Japan, Tokyo, Japan) for 60 min at 4°C at 100 V. Non-specific binding was blocked by incubation with 5% non-fat milk in Tris-buffered saline containing 0.1% Tween-20 (TBST) for 1 h at room temperature. After blocking, the membrane was incubated in primary antibody (1X PBST containing 1% milk, 1:2,000) overnight at 4°C. The membrane was then washed three times with PBST. The immunoblots were incubated for 1 h in a 1:10,000 dilution of goat anti-rabbit or anti-mouse IgG coupled with horseradish peroxidase as a secondary antibody (GE Healthcare Japan) in TBST containing 1% milk.

Finally, each protein was detected using an enhanced chemiluminescence detection system (ECL prime) and captured with an ImageQuant LAS 400 (both from GE Healthcare Japan). The antibody against EGFR was purchased from Santa Cruz Biotechnology, Inc. (Santa Cruz, CA, USA). Anti-phosphotyrosine antibody (4G10) was purchased from Merck Millipore (Darmstadt, Germany), and anti-β-actin antibody was purchased from Sigma-Aldrich Japan (Tokyo, Japan).

### Assessment of combination effect

A combination index (CI) was calculated using the Chou-Talalay method (25) and used to evaluate the combination effect of the two drugs. The CI quantitatively depicts synergism (CI<1), addictive effect (CI=1), and antagonism (CI>1).

## Results

### The characteristics of patients and efficacy of VNR + DIF and platinum-based chemotherapy

A total of 39 patients were included in this retrospective study. The ages of the 39 patients ranged from 35 to 84 years (median age, 65 years), with 16 males (41.0%) and 23 females (59%). All tumors were adenocarcinomas, and 31 patients had stage IV disease (79.5%). Seven patients received gefitinib prior to cytotoxic chemotherapy.

At the first cytotoxic chemotherapy, 24 patients received VNR + DIF chemotherapy (VNR + UFT, n=5; VNR + S-1, n=19) and the other 15 patients received platinum-based chemotherapy. Of the 15 patients in the platinum group, eight patients received CDDP-based chemotherapy (CDDP + gemcitabine, n=4; CDDP + docetaxel, n=4), and the seven others received carboplatin-based chemotherapy (carboplatin + paclitaxel, n=5; carboplatin + pemetrexed, n=1; carboplatin + gemcitabine, n=1).

Table I shows the patient characteristics according to the first-line chemotherapy regimen (VNR + DIF vs. platinum). There was no significant difference between the two regimen groups with regard to age, gender, disease stage, smoking status, EGFR mutation type, Eastern Cooperative Oncology Group (ECOG) performance status (PS), and chemotherapy line. As a later line of cytotoxic chemotherapy, seven (29.2%) patients in the VNR + DIF group received platinum-based chemotherapy, and four (26.7%) patents in the platinum group received VNR + DIF treatment.

**Figure 1:**
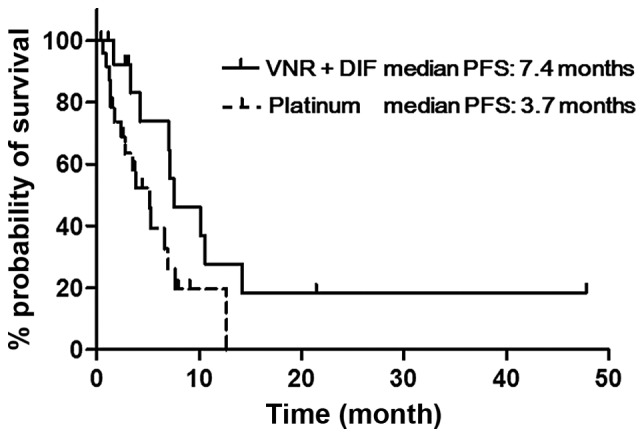
Kaplan-Meier curves of the progression-free survival (PFS) of the patients who received vinorelbine (VNR) + dehydrogenase-inhibitory fluoropyrimidine (DIF) chemotherapy (n=24) or platinum-based chemotherapy (n=15).

Both the objective response rate (ORR) and the disease control rate (DCR) of the VNR + DIF patients were favorable compared with those of the platinum group, although the differences were not significant (54.2 vs. 42.9%, p=0.74 and 87.5 vs. 71.4%, p=0.39; Table II). Fig. 1 shows the Kaplan-Meier curves for PFS in the VNR + DIF and platinum groups. The median PFS of the VNR + DIF group was significantly longer than that of the platinum group (7.4 vs. 3.7 months, p=0.02). The median OS was not significantly different between the two groups (36.6 vs. 35.4 months, p=0.34; Table II).

### The cell growth inhibition and effect of VNR, CDDP and 5-FU on EGFR phosphorylation in PC9 cells

Based on the results of the retrospective study, we speculated that VNR or DIF may have an effect on EGFR activity. To address this speculation, we performed *in vitro* experiments using PC9 cells harboring an active form of *EGFR* mutation.

**Figure 2:**
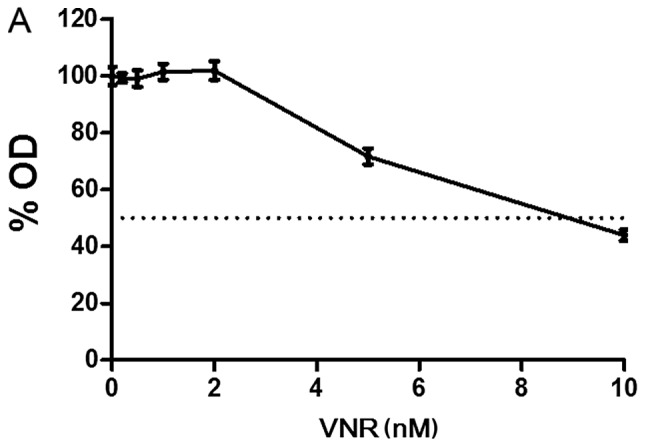

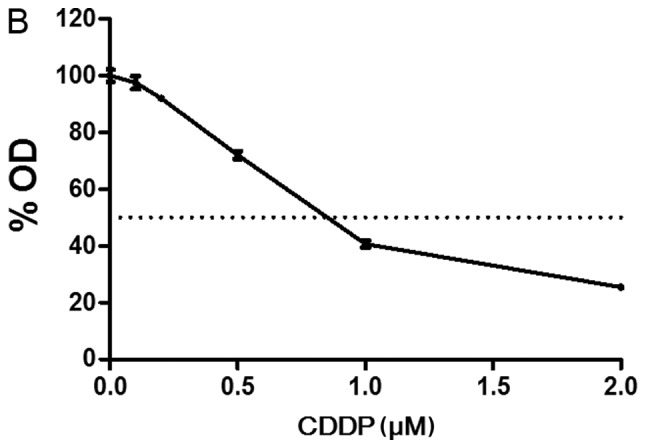

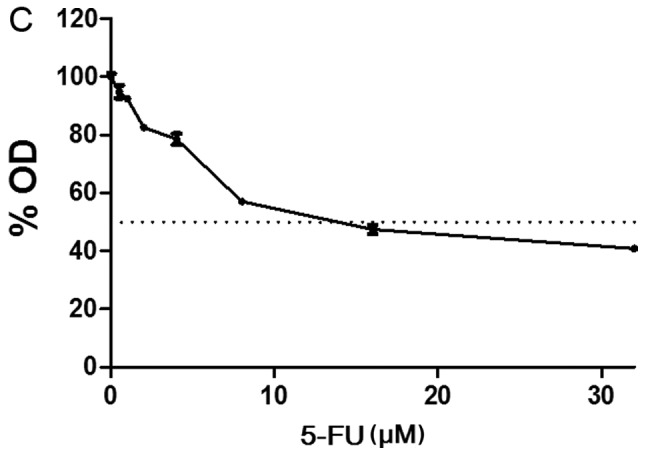
Sensitivity of PC9 cells to vinorelbine (VNR), cisplatin (CDDP), and 5-fluorouracil (5-FU). (A–C) PC9 cells were treated with the indicated concentrations of VNR, CDDP, or 5-FU for 24, 24 and 72 h, respectively. The survival cell fraction is expressed as the percentage of optical density (% OD) in reference to the OD of untreated cells using a 3-(4,5-dimethylhiazol-2-yl)-2,5-diphenyltetrazolium bromide (MTT) assay 72 h after the start of drug exposure. Data are means ± SD of three separate experiments.

We first evaluated the sensitivity of PC9 cells to VNR, 5-FU, and CDDP. The half-life of VNR in plasma after intravenous injection is ~20 h (26), and CDDP is almost completely eliminated within 24 h from plasma (27). In previous studies, DIF was orally administered to patients for 5 days in the combination of VNR + DIF (19,20), and the 5-FU concentration in plasma stayed roughly constant during an oral intake of DIF (28,29). We therefore exposed PC9 cells to VNR, CDDP and 5-FU for 24, 24 and 72 h, respectively, and 72 h after the start of drug exposure, we performed an MTT assay to evaluate the inhibition of cell proliferation. The concentration of VNR producing a 50% inhibition of cell growth (IC_50_) was 8.1 nM, that of CDDP was 0.59 μM, and that of 5-FU was 13.8 μM (Fig. 2), and these are clinically achievable concentrations (26–29).

**Figure 3:**
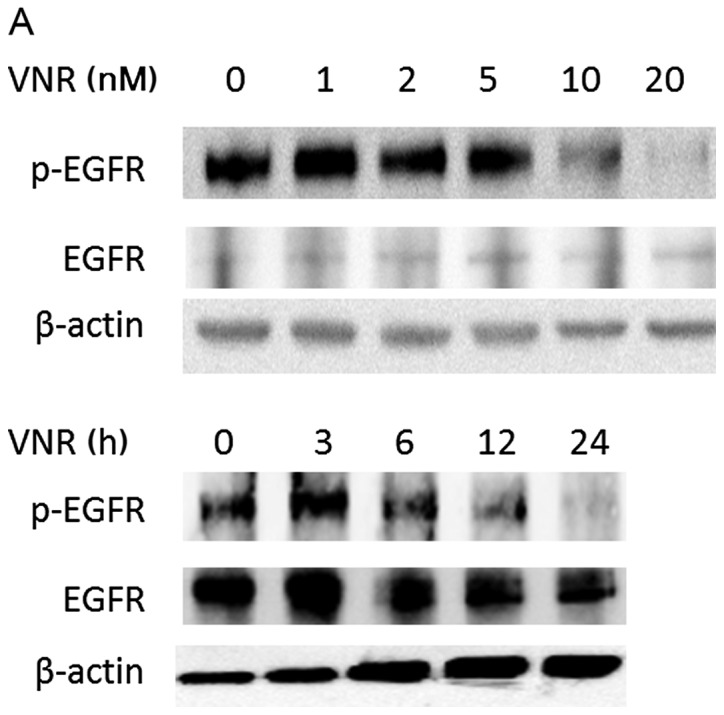

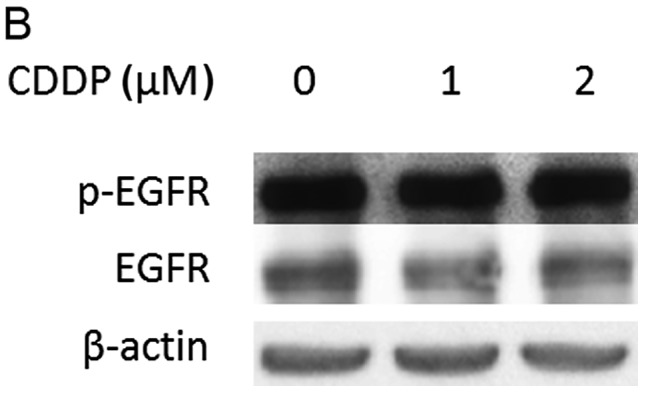

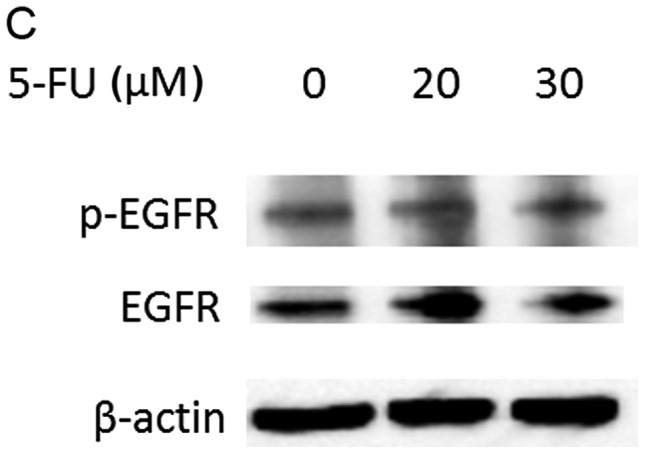
Effects of vinorelbine (VNR), cisplatin (CDDP), and 5-fluorouracil (5-FU) on epidermal growth factor receptor (EGFR) phosphorylation in PC9 cells. (A) PC9 cells were treated with the indicated concentrations of VNR for 24 h (upper panels), or 20 nM VNR for the indicated time (lower panels). Total cellular protein (1 mg) from cell lysate was immunoprecipitated using anti-EGFR antibody and subjected to a western blot analysis with anti-phosphotyrosine (p-EGFR, upper panel), and the membrane was stripped of bound antibodies and re-probed with anti-EGFR antibody (middle panel). Total cellular protein (20 μg) of the same lysate was subjected to a western blot analysis with β-actin (lower panel). (B and C) PC9 cells were treated with the indicated concentrations of CDDP or 5-FU for 24 h and processed as described above.

We evaluated the phosphorylation of EGFR after the treatment with each drug at the concentrations up to ~2-fold higher than the IC_50_. After the treatment with a 10 nM or higher concentration of VNR for 24 h, the phosphorylation of EGFR was clearly decreased. In the PC9 cells, this EGFR dephosphorylation induced by VNR appeared 12–24 h after the start of the exposure to 20 nM VNR (Fig. 3A), whereas such dephosphorylation of EGFR was not detected in the 24-h treatment with 5-FU or CDDP at the concentrations tested (Fig. 3B and C).

### The cell growth inhibition and effects of gefitinib, VNR, CDDP and 5-FU on EGFR phosphorylation in 1BR3-LR cells

To elucidate whether the suppression of EGFR phosphorylation induced by VNR functions as an anti-proliferative mechanism of VNR, we used 1BR3 cells (in which EGFR is not expressed), stably transfected with the L858R mutant *EGFR* (1BR3-LR).

**Figure 4:**
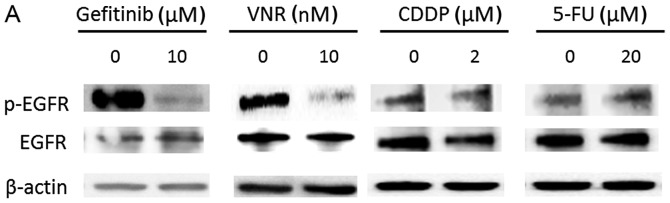

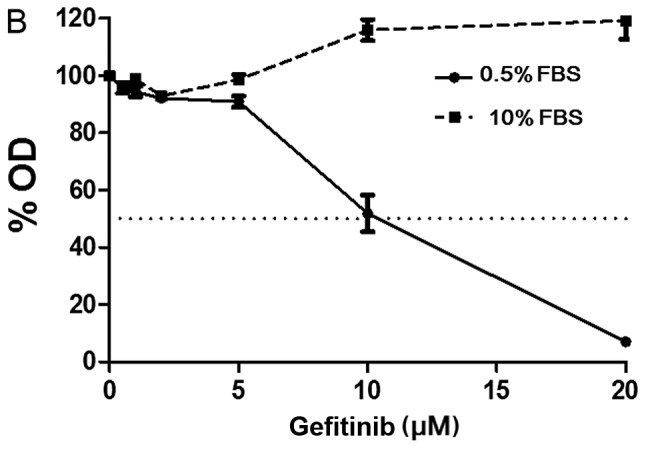

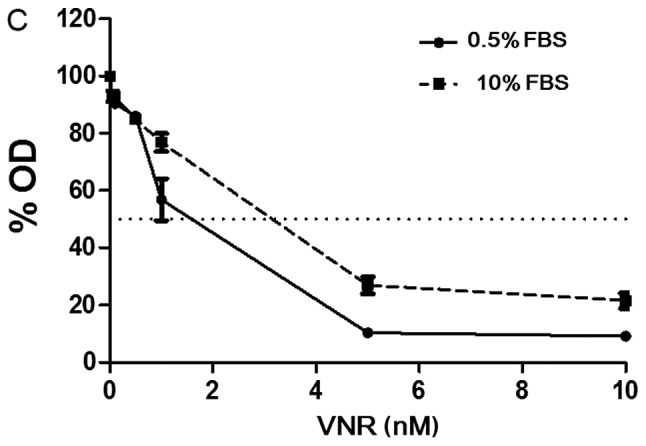

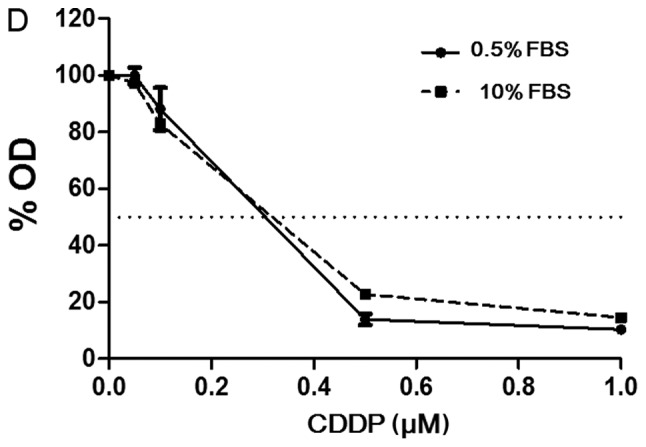

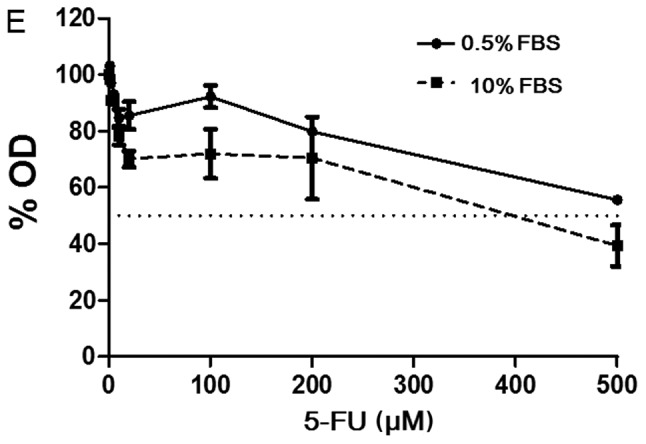
Effects of vinorelbine (VNR), cisplatin (CDDP), and 5-fluorouracil (5-FU) on epidermal growth factor receptor (EGFR) phosphorylation and cell growth inhibition in 1BR3-LR cells. (A) 1BR3-LR cells were treated with the indicated concentrations of gefitinib, VNR, CDDP, or 5-FU for 24 h. Total cellular protein (1 mg) from cell lysate was immunoprecipitated using anti-EGFR antibody and subjected to a western blot analysis with anti-phosphotyrosine (p-EGFR, upper panel), and the membrane was stripped of bound antibodies and re-probed with anti-EGFR antibody (middle panel). Total cellular protein (20 μg) of the same lysate was subjected to a western blot analysis with β-actin (lower panel). (B–E) 1BR3-LR cells were treated with the indicated concentrations of gefitinib, VNR, CDDP, or 5-FU for 72 h in the medium containing 10% (solid line) or 0.5% (dotted line) fetal bovine serum (FBS). The survival cell fraction is expressed as the percentage of optical density (% OD) in reference to the OD of the untreated cells in an 3-(4,5-dimethylhiazol-2-yl)-2,5-diphenyltetrazolium bromide (MTT) assay. Data are presented as means ± SD of three separate experiments.

We determined the effects of gefitinib, VNR, CDDP and 5-FU on EGFR phosphorylation in 1BR3-LR cells. As shown in Fig. 4A, the treatment with 10 nM VNR for 24 h suppressed EGFR phosphorylation as well as gefitinib did, a selective EGFR-TKI in 1BR3-LR cells. Similar to the PC9 cells, neither CDDP nor 5-FU induced the dephosphorylation of EGFR.

We evaluated the cell growth inhibition by these drugs in 1BR3-LR cells. In 1BR3-LR cells cultured in 10% FBS-containing medium, gefitinib slightly promoted cell growth, although it effectively suppressed EGFR phosphorylation. Gefitinib inhibited the cell growth concentration dependently in the medium containing 0.5% FBS (Fig. 4B), indicating that the proliferation or survival of 1BR3-LR cells is dependent on EGFR-mediated signaling in low-serum condition.

We compared the growth inhibitory activities of VNR, 5-FU, and CDDP in 1BR3-LR cells between normal (10%) and low (0.5%) serum conditions, and we found that the cell growth inhibition by VNR was enhanced in the low-serum condition compared to that in the normal-serum condition (Fig. 4C). The sensitivity of 1BR3-LR cells to CDDP did not clearly differ by serum concentration (Fig. 4D). In the low-serum condition, 1BR3-LR cells tended to be resistant to 5-FU-induced cell growth inhibition (Fig. 4E).

### The effect of Na_3_VO_4_ on EGFR phosphorylation and gefitinib- and VNR-induced cell growth inhibition

**Figure 5:**
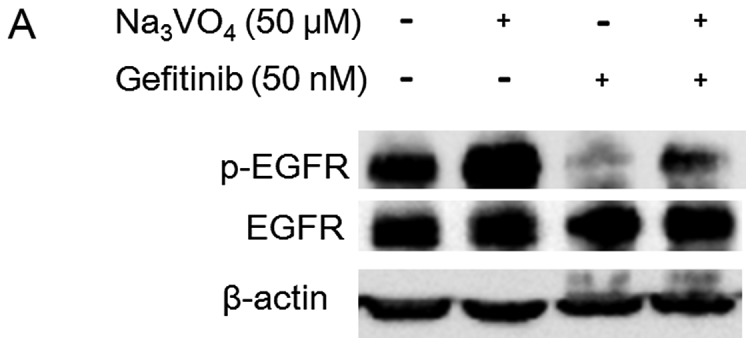

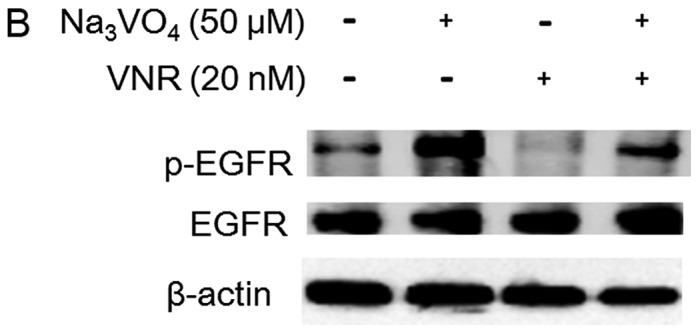

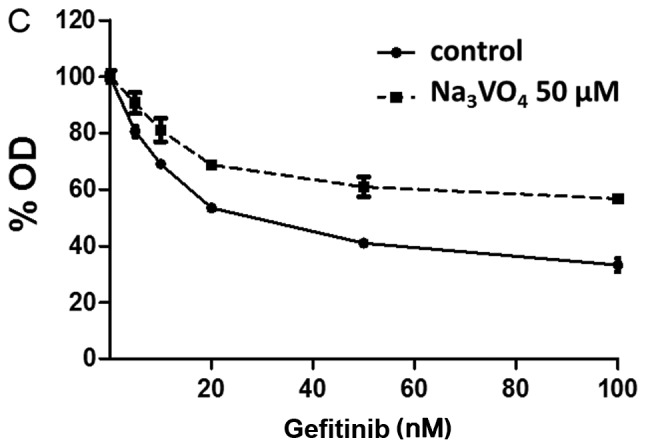

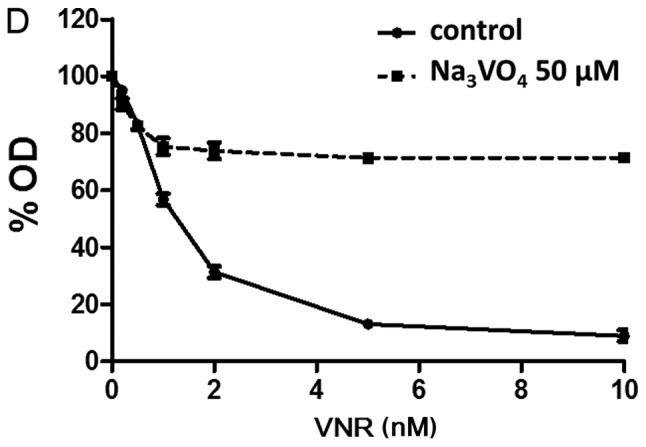
Effects of sodium orthovanadate (Na_3_VO_4_) on epidermal growth factor receptor (EGFR) phosphorylation and the cell growth inhibition by gefitinib or vinorelbine (VNR) in PC9 cells. (A and B) PC9 cells were treated with 50 nM gefitinib or 20 nM VNR in the presence or absence of 50 μM Na_3_VO_4_ for 24 h. Total cellular protein (1 mg) from cell lysate was immunoprecipitated using anti-EGFR antibody and subjected to a western blot analysis with anti-phosphotyrosine (p-EGFR, upper panel), and the membrane was stripped of bound antibodies and re-probed with anti-EGFR antibody (middle panel). Total cellular protein (20 μg) of the same lysate was subjected to a western blot analysis with β-actin (lower panel). (C and D) PC9 cells were treated with the indicated concentrations of gefitinib or VNR in the presence or absence of 50 μM Na_3_VO_4_ for 72 h. The survival cell fraction is expressed as the % OD in reference to the OD of the untreated cells in an 3-(4,5-dimethylhiazol-2-yl)-2,5-diphenyltetrazolium bromide (MTT) assay. Data are means ± SD of three separate experiments.

To further test whether the EGFR dephosphorylation induced by VNR was related to anti-proliferative effect of VNR, we tested whether Na_3_VO_4_, an inhibitor of protein tyrosine phosphatases, can interfere with the gefitinib- or VNR-induced dephosphorylation of EGFR and affect the cell growth inhibition by gefitinib or VNR in PC9 cells. We treated PC9 cells with 50 nM gefitinib or 20 nM VNR in the presence or absence of 50 μM Na_3_VO_4_ for 24 h and then evaluated the EGFR phosphorylation. The EGFR dephosphorylation caused by gefitinib or VNR was clearly inhibited in the presence of Na_3_VO_4_ (Fig. 5A and B).

The cell growth inhibition of PC9 cells by gefitinib or VNR was compared in the presence or absence of Na_3_VO_4_. As shown in Fig. 5C and D, the cell growth inhibitory activity of both gefitinib and VNR was greatly interfered with by Na_3_VO_4_.

### Synergistic cell growth inhibition by the combination of gefitinib or VNR with 5-FU in PC9 cells

In our previous study, the combination treatment of VNR and subsequent 5-FU synergistically inhibited cell growth in three NSCLC cell lines (18). In the present study, to reproduce this synergism and to clarify whether EGFR suppression by VNR is related to this interaction, we evaluated the combination effects using the CI and the simultaneous combination of gefitinib and 5-FU, or the sequential treatment of VNR followed by 5-FU. Since gefitinib suppressed EGFR activity within 1 h *in vitro* (6), gefitinib and 5-FU were combined simultaneously.

**Figure 6:**
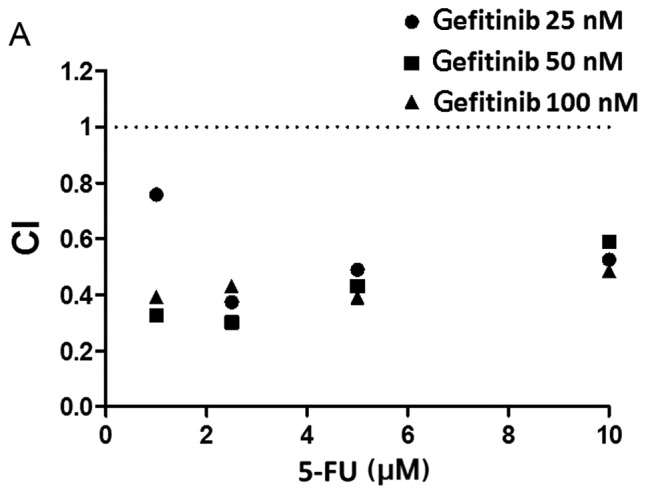

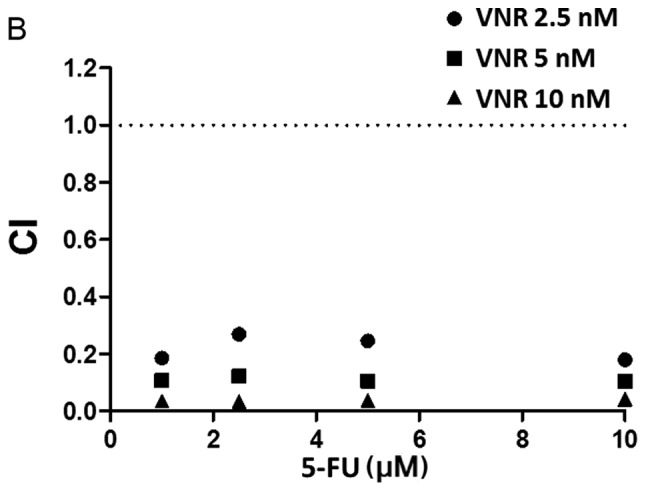
The synergistic cell growth inhibition by the combination of gefitinib or vinorelbine (VNR) with 5-fluorouracil (5-FU) in PC9 cells. (A) PC9 cells were treated with either a single agent or the simultaneous combination of 5-FU and gefitinib for 72 h. (B) PC9 cells were treated with either a single agent or the sequential combination of VNR for 24 h and 5-FU for the next 72 h. The viabilities of the cells were determined in an 3-(4,5-dimethylhiazol-2-yl)-2,5-diphenyltetrazolium bromide (MTT) assay. The combination index (CI) for each concentration of 5-FU was calculated by the Chou-Talalay method.

We treated PC9 cells with the indicated concentrations of gefitinib + 5-FU for 72 h or VNR for 24 h and 5-FU for the following 72 h, and we calculated the CI (Fig. 6). As shown in the Fig., the CI values for the combination of gefitinib and 5-FU were all <1.0, indicating that this simultaneous combination showed synergistic cell growth inhibitory activity against PC9 cells. Similar results were achieved for sequential exposure to VNR followed by 5-FU with CI<0.3, which implied strong synergism (Fig. 6B).

## Discussion

The aim of this study was to evaluate whether the combination of VNR + DIF is a more effective treatment compared with the standard platinum-based chemotherapy in *EGFR*-mutated lung adenocarcinoma patients, and then to clarify the underlying mechanism by which VNR + DIF was efficacious in such patients. In the retrospective analysis, the PFS of the patients who received VNR + DIF chemotherapy was longer than that of the patients who received platinum-based chemotherapy. Using mutated EGFR-expressing cells, we found that VNR induced EGFR dephosphorylation and that this effect of VNR may be related to its cell growth inhibitory activity. We propose that EGFR inhibition by VNR may be one of the mechanisms of the synergistic effect by the sequential treatment of VNR and subsequent 5-FU.

In this retrospective study, the characteristics of the patients who received VNR + DIF chemotherapy were not significantly different from those who received platinum-based chemotherapy. Nevertheless, the PFS of the VNR + DIF treatment group was significantly longer than that of the platinum-based chemotherapy group. The RR and DCR values of the VNR + DIF chemotherapy patients also tended to be better than those of the platinum-based chemotherapy patients, although the difference was not significant. These results suggest that the combination of VNR + DIF may be more effective than platinum-based chemotherapy, at least in terms of the antitumor effect in lung adenocarcinomas with EGFR-activating mutations.

Despite the significant difference in PFS, the OS of the present two regimen groups was not significantly different. Over one-quarter of the patients in each group crossed over to the other regimen as a later-line treatment. The comparison of OS was performed between small groups (n=24 for VNR + DIF, n=15 for platinum), and thus the statistical power was low. We suspect that the lack of a significant difference in OS was due to these reasons. In a proportional hazard analysis performed in another study, we found that the application of the VNR + DIF combination but not platinum-based chemotherapy was a significant and independent factor to prolong survival in lung adenocarcinoma patients with *EGFR* mutations (unpublished data). These results suggest that VNR + DIF chemotherapy may be superior to platinum-based chemotherapy in the treatment of lung adenocarcinoma patients with *EGFR* mutations.

To clarify the mechanisms by which VNR + DIF chemotherapy was favorable in the treatment of *EGFR*-mutated lung adenocarcinoma, we focused on the effects of VNR and 5-FU on EGFR phosphorylation. In *EGFR*-mutated PC9 cells, VNR induced EGFR dephosphorylation 12–24 h after drug exposure at the concentration of 10 nM or higher. In the treatment of NSCLC, when 20–30 mg/m^2^ of VNR is administered, a VNR concentration >10 nM is maintained in peripheral blood for 12–24 h (26). Thus, an EGFR-dephosphorylating concentration of VNR is clinically achievable.

The sufficiently cell growth-inhibiting and clinically relevant concentration of CDDP and 5-FU (27–29) did not affect the EGFR phosphorylation in PC9 cells. Our observation in terms of EGFR dephosphorylation by VNR is in accord with the result of a previous investigation. Wu *et al* reported that in esophageal cancer cells, the disruption of the microtubule network induced by microtubule-targeting drugs such as docetaxel and vincristine, another vinca-alkaloid, was associated with EGFR dephosphorylation and the subsequent inhibition of Akt and Erk (30). VNR is a semisynthetic vinca-alkaloid, a member of the family of microtubule-targeting drugs. Although the precise mechanism is still unknown, EGFR-suppressing activity may thus be a common property among taxanes and vinca-alkaloids.

To test whether the EGFR dephosphorylation induced by VNR is associated with its anti-proliferative effect, we took advantage of 1BR3-LR cells, which express an active form of EGFR. Parental 1BR3 cells do not express EGFR. Although we observed that 1BR3-LR cells were completely resistant to gefitinib in normal culture medium containing 10% FBS, gefitinib showed cell growth inhibition against 1BR3-LR cells in the medium containing 0.5% FBS. These results indicate that the growth or survival of 1BR3-LR cells is at least partially dependent on EGFR signaling in a low-serum condition.

We also found that the growth inhibition of 1BR3-LR cells by VNR was enhanced in the low-serum condition, although such changes of drug sensitivity were not observed in CDDP- or 5-FU-treated cells. These findings strongly support the interpretation that the enhanced sensitivity to VNR in the low-serum condition is not a non-specific effect but rather is due to the suppression of EGFR signaling, since both gefitinib and VNR (and not CDDP or 5-FU) suppressed EGFR phosphorylation.

This interpretation is further supported by our finding that Na_3_VO_4_ interfered with the EGFR dephosphorylation induced by gefitinib and VNR, and suppressed the cell growth inhibition by these agents in PC9 cells. Taken together, our results led us to conclude that VNR-induced EGFR dephosphorylation is associated with the anti-proliferative effect of VNR in lung adenocarcinoma cell lines harboring *EGFR* mutations.

We found previously that the combination of VNR followed by 5-FU resulted in synergistic cell growth inhibition in three NSCLC cell lines (18). The synergism was also observed in PC9 cells harboring an *EGFR* mutation with the sequential treatment of VNR and then 5-FU. Therefore, although it still remains to be determined whether the EGFR suppression by VNR itself may lead to a better antitumor effect of VNR in *EGFR*-mutated lung adenocarcinoma, it is possible that this synergism also contributed to the favorable antitumor activity observed in patients treated with VNR + DIF.

In addition, as in an earlier study (31), the simultaneous combination of gefitinib and 5-FU showed synergistic cell growth inhibition in PC9 cells in the present study. Therefore, the synergism of VNR followed by 5-FU may be attributable, at least in part, to the EGFR-suppressing activity of VNR.

The important therapeutic target of 5-FU is thymidylate synthase (TS), and the downregulation of TS would be expected to enhance the cytotoxicity of 5-FU (32). EGFR signal transduction has been shown to be involved in the expression of *TS* genes (33,34), and in our previous study, VNR as well as gefitinib was shown to suppress TS expression (18). Thus, the decrease of TS caused by EGFR suppression may be a common mechanism of the synergism by the combination of VNR or gefitinib with 5-FU.

The identification of activating mutations of the *EGFR* gene in a subset of NSCLC patients led to a change in the treatment of the disease (6), and the presence of *EGFR* mutations is a predictive marker of response to EGFR-TKI (3,4). It has been reported that the effect of cytotoxic chemotherapy is not different between patients with and without *EGFR* mutations (35,36). Thus, the cytotoxic agents for NSCLC patients with *EGFR* mutations are not different from those used for *EGFR* wild-type patients. To our knowledge, there has been no prospective study attempting to identify which agents or combination chemotherapy is specifically effective in *EGFR*-mutated NSCLC.

The identification of such cytotoxic agents or combination chemotherapy is expected to improve the survival of NSCLC patients harboring *EGFR* mutations. In the present study, we observed favorable PFS by the combination of VNR + DIF and the potential mechanism of this good treatment outcome. We propose that the combination chemotherapy of VNR and DIF can be a promising strategy for NSCLC patients harboring *EGFR* mutations. Since our observations were retrospective and experimental, there are several limitations. To establish the optimal VNR + DIF combination chemotherapy in NSCLC patients with *EGFR* mutations, we are performing a prospective phase II trial of this treatment targeting such patients.

In conclusion, the PFS afforded by the VNR + DIF combination treatment was significantly longer compared to that of platinum-based chemotherapy in lung adenocarcinoma patients with *EGFR* mutations. VNR suppressed EGFR phosphorylation in PC9 cells, and this activity may be related with cell growth inhibition of VNR, and the synergistic cell growth inhibition when VNR was combined with 5-FU. The combination chemotherapy of VNR + DIF may be a promising treatment for NSCLC patients with *EGFR* mutations.

## Figures and Tables

**Table I:** Characteristics of the 39 lung adenocarcinoma patients harboring *EGFR* mutations.

	VNR + DIF (n=24)	Platinum (n=15)	P-value
Age (years)			0.31^a^
Median (range)	66.5 (50–84)	64 (35–74)	
Sex			0.92^b^
Male	10	6	
Female	14	9	
Disease stage			0.84^c^
IIIA	1	0	
IIIB	3	2	
IV	19	12	
Recurrence	1	1	
Histology			
Adenocarcinoma	24	15	
Smoking status			0.74^c^
Current	3	3	
Former	5	4	
Never	16	8	
EGFR mutation type			0.41^c^
Exon 19 deletion	13	6	
Exon 21 point mutation	7	7	
Minor mutation	2	0	
Unknown	2	2	
Performance status			0.44^c^
0	14	8	
1	10	6	
2	0	1	
Chemotherapy line			0.87^b^
First-line	20	12	
Second-line (gefitinib as first-line)	4	3	

a Mann-Whitney test

b Fisher’s exact test and

c χ^2^ test.

EGFR, epidermal growth factor receptor; VNR, vinorelbine; DIF, dihydropyrimidine dehydrogenase-inhibitory fluoropyrimidine.

**Table II:** Comparison of efficacy parameters between the combination of VNR + DIF and platinum-based chemotherapy.

Confidence interval (95%)	VNR + DIF (n=24)	Platinum (n=15)	P-value
ORR	54.2 (32.0–76.4)	42.9 (29.6–56.1)	0.74^a^
DCR	87.5 (80.7–94.3)	71.4 (59.4–83.5)	0.39^a^
mPFS (months)	7.4 (6.2–8.7)	3.7 (2.9–4.6)	0.02^b^
mOS (months)	36.6 (27.2–46.0)	35.4 (31.0–39.7)	0.34^b^

a Fisher’s exact test and

b log-rank test.

VNR, vinorelbine; DIF, dihydropyrimidine dehydrogenase-inhibitory fluoropyrimidine; ORR, objective response rate; DCR, disease control rate; mPFS, median progression-free survival; mOS, median overall survival.
